# Removal of Humic Acid Using 3-Methacryloxypropyl Trimethoxysilane Functionalized MWCNT Loaded TiO_2_/PES Hybrid Membrane

**DOI:** 10.3390/membranes11090721

**Published:** 2021-09-21

**Authors:** Noor Fazliani Shoparwe, Lim-Cee Kee, Tunmise Ayode Otitoju, Hafiza Shukor, Nor’Izzah Zainuddin, Muaz Mohd Zaini Makhtar

**Affiliations:** 1 Faculty of Bioengineering and Technology, Jeli Campus, Universiti Malaysia Kelantan, Jeli 17600, Kelantan, Malaysia; fazliani.s@umk.edu.my (N.F.S.); getcheeked@gmail.com (L.-C.K.); timopd1@gmail.com (T.A.O.); 2 School of Materials Science and Engineering, Shenyang University of Technology, Shenyang 110870, China; 3 Centre of Excellence For Biomass Utilization (CoEBU), Faculty of Chemical Engineering Technology, Universiti Malaysia Perlis, Arau 02600, Perlis, Malaysia; hafizashukor@unimap.edu.my; 4 Indah Water Konsortium, Lorong Perda Utama 13, Bukit Mertajam 14300, Pulau Pinang, Malaysia; izzahniz@yahoo.com; 5 Bioprocess Technology Division, School of Industrial Technology, Universiti Sains Malaysia, Gelugor 11800, Pulau Pinang, Malaysia; 6 Fellow of Center for Global Sustainability Studies, Universiti Sains Malaysia, Gelugor 11800, Pulau Pinang, Malaysia

**Keywords:** polyethersulfone, mixed matrix membrane, humic acid

## Abstract

In the present work, a highly efficient mixed matrix membrane (MMM) for humic acid (HA) removal was developed. Multiwalled carbon nanotubes (MWCNTs) were functionalized in the presence of 3-methacryloxypropyl trimethoxysilane using the co-condensation method and were subsequently loaded with TiO_2_ (prepared via the sol–gel route). The as-prepared material was then incorporated into a PES polymer solution to prepare a fMWCNT-TiO_2_/PES hybrid membrane via non-solvent induced phase inversion. The microstructure of the membrane was characterized using Fourier transform infrared spectroscopy, atomic force microscopy, scanning electron microscopy, water contact angle, thickness, porosity, and pore size. The fMWCNT-TiO_2_/PES hybrid membrane was tested for the removal of HA and antifouling performance. The results show that the surface hydrophilicity of the membranes was greatly improved upon the addition of the fMWCNT-TiO_2_ particles. The results show that 92% of HA was effectively removed after 1 h of filtration. In comparison with pristine membrane, the incorporation of fMWCNT-TiO_2_ nanoparticles led to enhanced pure water flux (99.05 L/m^2^ h), permeate flux (62.01 L/m^2^ h), higher HA rejection (92%), and antifouling improvement (RFR: 37.40%, FRR: 86.02%). Thus, the fMWCNT-TiO_2_/PES hybrid membrane is considered to be a great potential membrane for the improvement of ultrafiltration membranes.

## 1. Introduction

In the past, numerous approaches have been the main focus of scientific research for the removal of natural organic matter (NOM), including humic acid (HA), in natural waters and soil. HA is derived from the decay in plants, animals, and other biological activities of microorganisms in the environment [1]. The presence of HA in water gives an undesirable taste and color, providing nutrient nutrition for the growth of bacteria, which is unfavourable for water quality. Furthermore, HA may react with chlorine disinfectant during drinking water treatment, producing a hazardous by-product that is detrimental to the reproductive, human nervous, renal, and circulatory systems. On the other hand, HA can intensify microbial regrowth in water distribution networks [2]. Presently, it is difficult to treat water for HA using conventional treatment processes. Currently, the approaches used to reduce or eliminate HA include coagulation–flocculation [3], filtration [4], biological treatments [5,6], advanced oxidation technology [7,8], adsorption [9,10], and membrane separation [11]. Among the aforementioned methods, membrane separation is simple and efficient. Polymeric membranes are a crucial component for technological membrane processes and are limited due to fouling. The surface of the membrane becomes intensely fouled, leading to flux decline, lifespan reduction, and high operational costs [12]. Therefore, the development of highly efficient membranes for HA removal from aqueous solutions has become the research focus for drinking water treatment.

Polyethersulfone, polysulfone, polypropylene, polyvinylidene fluoride, and polytetrafluoroethylene are often used to fabricate polymeric membranes due to their outstanding performance. Polyethersulfone (PES) has been widely employed to prepare membranes for water applications. Among other polymers, PES has outstanding properties, which include high thermal, mechanical stability, and chemical resistance as well as outstanding oxidative properties, thus making PES an ideal polymer to fabricate an asymmetric membrane with different pore structures and radii [13]. However, this performance is threatened due to pore-clogging from solute adsorption on the surface of the membrane, thereby leading to poor separation efficiency. To reduce this phenomenon, many attempts or methods, including chemical treatment, surface coating, and blending (with organic or inorganic materials), have received increasing attention. An approach for incorporating inorganic or nanoparticles (NPs) into polymer-doped solutions has been highly favored in recent years due to its simple preparation and operational process. A significant number of works have reported on the use of different NPs additives including ZnO, SiO_2_, Al_2_O_3_, CNTs, ZrO_2_, and TiO_2_. Modifying PES with the aforementioned particles has been widely explored to improve the mechanical strength, permeability, hydrophilicity, effective surface structure control, and solute rejection of membranes. However, mixed matrix membranes suffer from inherent drawbacks. When NPs with a low specific surface area are dispersed in a polymer-doped solution, the high content will induce particle aggregation, causing a defect in pore structure and a reduction in modification effects [14]. Therefore, it is necessary to redesign the NPs structure (with a high specific surface) as an inorganic additive in order to lower the additive proportion.

Multi-walled carbon nanotubes (MWCNTs) have received a great deal of attention due to their outstanding properties, including chemical stability, high specific surface area, thermal conductance, and easy functionalization [15]. MWCNTs tends to create a rough surface at the nano/micrometer level due to its rigid cylindrical nanostructures, which have the potential of leading to an increase in membrane permeability, filtration area, mechanical strength, and antifouling property [16] and can also serve extraordinarily as channels to mass transport channels [17]. Considering this fact, the fouling of the membrane poses a serious problem, particularly during water reclamation, because MWCNTs present a high solute adsorption capacity. Despite many their advantages, unmodified MWCNTs can form aggregates via Van der Waals forces, and the chemically inert nature of MWCNTs generally contributes to weak interfacial interactions and poor dispersibility within the polymer matrix [18]. The dispersing ability of MWCNTs in polymer solutions has a vital effect on performance and antifouling properties; therefore, the modification of MWCNTs is of significant importance. Up until now, various methods have been widely used to modify MWCNTs, which include the attachment of polar groups to the sidewalls of MWCNTs, the introduction of functional groups on the surfaces of MWCNTs, and functionalizing with chemical agents [19]. Table 1 shows a summary of the investigations on functionalized MWCNTs/PES membranes.

As mentioned above, the unmodified MWCNTs usually suffer from self-aggregation due to their intrinsic Van der Waals forces [34]. Furthermore, due to the presence of catalytic impurities and surface defects, interfacial bonding with functional groups tends to be limited [35]. The effective and efficient utilization of functionalized MWCNTs will strongly depend on its ability to disperse homogeneously as well as to achieve effective interfacial bonding [36]. To this end, the development of improved MWCNTs is of significant necessity. In this work, the MWCNTs were treated with 3-methacryloxypropyl trimethoxysilane (MPS) to make them more reactive and to enable better dispersion [37]. To the best of our knowledge, TiO_2_ loaded MPS-functionalized multiwalled carbon nanotubes have not yet been introduced as additives for PES membranes. The combination of functionalized MWCNTs (fMWCNTs) could create active sites, and the excited electron band of TiO_2_ could migrate into the MWCNTs as well as enhance homogenous dispersion in polymer-doped solutions without aggregation and could also improve the antifouling properties of MMM. To this end, MWCNTs are functionalized with MPS and are subsequently loaded with TiO_2_ (prepared via sol-gel synthetic route). The prepared particles were then incorporated into the PES-doped solution to produce the mixed matrix membrane. The TiO_2_ loaded fMWCNT/PES hybrid membrane with antifouling properties was tested for its effectiveness in rejecting HA from H_2_O. For comparison, the HA removal capabilities of the neat membrane, unfunctionalized membranes (using TiO_2_, MWCNTs), and the functionalized membranes (fTiO_2_, fMWCNTs) were also investigated under similar conditions.

## 2. Materials and Methods

### 2.1. Materials

PES was obtained from BASF Chemical Co. (Ludwigshafen, Germany). Multiwall carbon nanotubes (MWCNTs) were supplied by Nanocyl SA. (Sambreville, Belgium). The 3-methacryloxypropyl trimethoxysilane (MPS) and titanium tetraisopropoxide (TTIP, 97%) were provided by Wego Chemical Group (New York, NY, USA). N-N-dimethylacetamide (DMAc) and humic acid (HA) were kindly obtained from Sigma Aldrich (St. Louis, MO, USA). Toluene, ethyl alcohol, acetone, HCl, and NaOH were obtained from Merck (Darmstadt, Germany). Deionized (DI) H_2_O was obtained from water purification system, (Synergy System, Merck). Distilled H_2_O was obtained from our UMK laboratory. Nitrogen gas was obtained from Wellgas Sdn. Bhd. (Simpang Ampat, Malaysia). The PES was oven-dried at 80 °C for 5 h before use.

### 2.2. Synthesis of TiO_2_ Powder

TiO_2_ powder was purchased from Sigma-Aldrich (St. Louis, MO, USA) (Particle size: ≤25 nm, surface area: 45–55 m^2^/g) and was prepared according to the sol–gel synthetic route. In brief, ethyl alcohol (55 mL) was mixed with DI H_2_O (135 mL) and HCl (0.2 mL). Subsequently, TTIP (18.5 mL) was introduced dropwise into the mixture and was stirred continuously for 50 h using a magnetic stirrer. Thereafter, the obtained precipitate was filtered and washed with DI H_2_O and was thereafter dried for 23 h at 100 °C and calcined for 2.5 h at 510 °C. Finally, the sample was gently ground with a pestle and mortar to obtain titanium dioxide powder.

### 2.3. Synthesis of Functionalized TiO_2_ (fTiO_2_) or MWCNTs (fMWCNTs)

The fTiO_2_ or fMWCNTs particles were prepared using the co-condensation method. In brief, TiO_2_ or MWCNTs (1 g) was mixed with MPS (9 g) in toluene (1:9 *v*/*v*) and was stirred continuously for 6 h at 105 °C under reflux conditions. Subsequently, the reaction was filtered and cleaned using acetone to remove any MPS remnants. Finally, the fTiO_2_ or fMWCNTs were oven-dried for 6 h at 105 °C. 

### 2.4. Synthesis of TiO_2_ Functionalized MWCNTs (fMWCNTs-TiO_2_)

fMWCNTs-TiO_2_ was prepared by mixing fMWCNTs (1 g) with TiO_2_ (1 g) in the ratio of 1:1 under reflux condition at 105 °C for 6 h. Then, the obtained fMWCNTs-TiO_2_ was filtered and cleaned using DI water. Finally, the NPs were oven-dried for 12 h at 80 °C. 

### 2.5. Membrane Preparation

The membranes were prepared via non-solvent-induced phase inversion. The composition of the doped solutions for the prepared membranes is summarized in Table 2. In brief, a pre-determined amount of nanoparticles (TiO_2_, fTiO_2_, MWCNTs, fMWCNTs, or fMWCNTs-TiO_2_) were mixed in DMAc for 5 h. Subsequently, the polymer (PES) was introduced and dissolved in the solution under continuous agitation for about 18 h at 65 °C. The doped solution was sonicated for 2 h to eliminate any air bubbles. Finally, the doped solution was spread onto a clean glass plate with a casting blade that was 250 µm. Immediately, the plate with the polymer film was directly submerged in distilled H_2_O at 15 ± °C for 12 h to enable the exchange of the non-solvent and solvent. Finally, the membrane was dried for 24 h between two filter papers.

### 2.6. Membrane Characterization 

Fourier transform infrared spectroscopy (FTIR) analysis was observed using a Thermo Scientific™ Nicolet™ iS™10 FT-IR Spectrometer, powered by OMNIC Spectra software (Thermo Nicolet, Waltham, MA, USA). The analysis was conducted at a scanning range from 4000–425 cm^−1^ and was collected using 32 scans and a 4 cm^−1^ resolution. The surface images of the membrane were observed using a scanning electron microscope (SEM, TM3000, Hitachi, Tokyo, Japan). The membranes were mounted vertically on a double-sided carbon tape in order to hold the sample. In order to prevent electrostatic loading, the membranes were platinum sputtered under a vacuum at 15 kV. The thermal stability of the membrane was evaluated by means of thermogravimetric analysis using a TGA 7 Thermogravimetric Analyzer (Waltham, MA, USA). The membranes were heated from 30 °C to 800 °C at a constant heating rate of 20 °C/min under a nitrogen atmosphere. Membrane roughness was determined using atomic force microscopy (Park Scientific model XE-100, Park Systems Corp., Suwon, Korea). The sample of the membrane was mounted on a microscopic slide and was scanned at a scan size of 10 µm × 10 µm and a scan rate of 0.25 Hz under tapping mode. A contact angle goniometer (Model: OCA15plus, DataPhysics Instruments GmbH, Filderstadt, Germany) was used to characterize the hydrophilicity of the membrane at room temperature using deionized water. The membrane sample was mounted on a glass slide with double-sided tape, and a H_2_O droplet (0.2 µL) was dropped on the surface at room temperature using a motor-driven microsyringe. Subsequently, the water contact angle (WCA) between the membrane and the H_2_O droplet was analysed using DROP image software. In order to minimize experimental error, the average WCA value for each membrane was calculated using at least ten different locations. For membrane porosity measurements, the membranes were immersed in deionized H_2_O for 24 h at 25 °C. The membrane in the wet state was weighed and was subsequently oven-dried at 25 °C for 10 h. The porosity was determined using Equation (1):

(1) $$\varepsilon \left(\%\right)=\frac{W_{w}-W_{d}}{\left(W_{w}-W_{d}\right)/d_{w}+W_{d}/d_{p}}\times 100\;\%\;$$

where $W_{w}$, $W_{d}$, $d_{p}$, $d_{w}$, and ε refer to the wet membrane weight (g), dry membrane weight (g), PES density (1.37 g/cm^3^), the density of H_2_O (1.0 g/cm^3^), and membrane porosity, respectively.

The mean pore radius ($r_{m}$) was calculated using the Guereout–Elford–Ferry equation (Equation (2)), which is determined according to the results obtained from the membrane porosity and the pure water flux (PWF):

(2) $$r_{m}=\sqrt{\frac{\left(2.9-1.75Porosity\right)8\eta lQ}{Porosity\times A\times ∆P}}.\;$$

where *l* refers to membrane thickness (m), *η* denotes the H_2_O viscosity ($8.9\times 10^{-4}$ Pa·s), *Q* denotes the PWF (m^3^/s), ∆*P* denotes the operating pressure (0.2 MPa), and *A* denotes membrane filtration area (m^2^).

### 2.7. Membrane Performance

#### 2.7.1. Synthesis and Analysis of HA Solution

A HA aqueous solution was prepared by dispersing HA (50 mg) in DI H_2_O (1 L). The solution was sonicated for 1 h, and the pH was controlled at 7.7 using 1 M of NaOH and 1 M of HCl. The concentration of the HA in the permeate and feed was measured using a UV spectrophotometer (Cary 60 UV-Vis, Agilent Technologies, Santa Clara, CA, USA) with a wavelength of 254 nm.

#### 2.7.2. Membrane Permeation Test for HA Removal

Membrane performance was investigated according to PWF, HA flux (HAF), and HA rejection. Initially, the membrane was pressurized at 0.25 MPa for 25 min, and then the pressure was reduced to 0.2 MPa. The pure water flux ($J_{WF}$) was determined using Equation (3). In the following step, the feed liquid was changed to the foulant (HA) solution. The flux for the HA solution was measured at the same pressure (0.2 MPa) for 1 h and was named $J_{HA}$. The HAF, HA rejection, and relative flux reduction (*RFR*) were measured using Equations (4)–(6), respectively.

(3) $$J_{WF}=\frac{V}{A_{m}t}\;\;\;$$

(4) $$J_{HA}=\frac{V}{A_{m}t}$$

(5) $$R=\left(1-\frac{C_{p}}{C_{f}}\right)\times 100\%$$

(6) $$RFR=\left(1-\frac{J_{HA}}{J_{WF}}\right)\times 100\%\;$$

where *R* denotes HA rejection (%), *RFR* denotes relative flux reduction (%), $C_{p}$ denotes the HA concentration (in permeate), $C_{f}$ denotes the HA concentration (in feed), $J_{WF}$ denotes the PWF (L/m^2^ h), $J_{HA}$ denotes the HAF (L/m^2^ h), V denotes permeate volume (L), t denotes filtration time (h), and $A_{m}$. denotes the effective filtration area (m^2^).

At the end of HA solution filtration, the membranes were thoroughly washed with deionized H_2_O, and then the PWF of the cleaned membrane ($J_{WF2}$) was measured. Then, the relative flux recovery ratio (*FRR*) was determined using Equation (7).

(7) $$FRR=\frac{J_{WF2}}{J_{WF}}\times 100\%$$

where *FRR* denotes the relative flux recovery ratio (%).

## 3. Result and Discussion

### 3.1. Membrane Characterization

**Figure 1:**
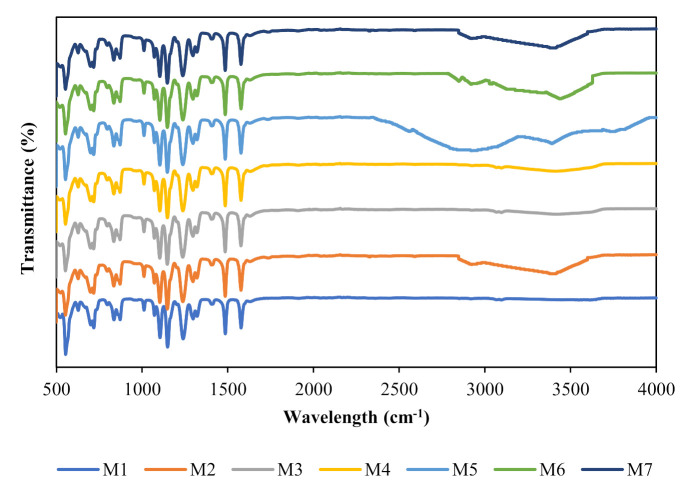
Fourier transform infrared spectroscopy (FTIR) spectra of the pristine membrane and composite membranes.

Figure 1 shows the IR spectra of the pristine and composite membranes. As observed, the peaks of all of the composite membranes differ from the pristine membrane. For M3 and M4, the spectrum showed no appreciable changes in the chemical structure, indicating zero molecular interaction between the NPs and PES. The broad peak at 3422 cm^−1^ can be ascribed to the hydroxyl groups, and the two peaks at 2851 cm^−1^ and 2922 cm^−1^ can be attributed to the C-H groups. The overlapping peaks at 1030 cm^−1^ and 1642 cm^−1^ correspond to the stretching vibration of Si-O-C and the asymmetric stretching vibration of the vinyl groups. The FTIR spectra of the functionalized membranes did not reveal any significant difference from that of the unfunctionalized membranes.

**Figure 2:**
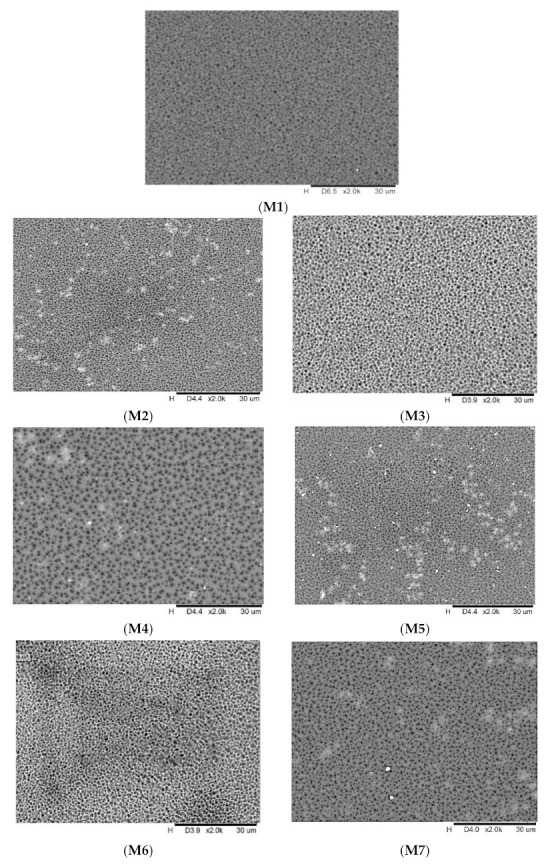
Surface images of M1, M2, M3, M4, M5, M6 and M7 membrane at ×2000 magnification.

The surface morphology of the membranes is presented in Figure 2. As clearly seen in the SEM micrograph, the white clusters confirm the existence of NPs on the membrane surface. As observed, the membranes prepared with functionalized particles (M5–M7) present less agglomerates than the membranes incorporated with unmodified nanoparticles (M2–M4). This could be the result of the existence of functional groups (carboxyl groups) in the NPs, which facilitate their good dispersion within the matrix. The high aggregation tendency of the M2–M4 membranes could be due to the high surface free energy of the nanoparticles. Among all of the composite membranes, the M7 membrane has a relatively smoother surface with less agglomeration than the other MMMs. This could be the result of better dispersion as a consequence of the bonding between the epoxy groups in the fMWCNTs and the hydroxyl groups in the TiO_2_. Hypothetically, the presence of TiO_2_ NPs will act as a spacer and prevent the agglomeration of the MWCNTs and will thus generate new hybrid NPs. As confirmed by Kedem et al. [38], TiO_2_ has a strong affinity toward the hexagonal planar skeleton of the carbon atoms in MWCNT.

**Figure 3:**
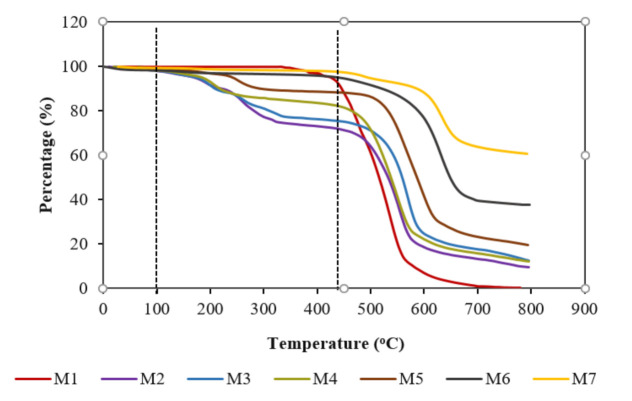
TGA graph of pristine membrane (M1) compared to the non-functionalized membranes (M2, M3, and M4) and the functionalized membranes (M5, M6 and M7).

TGA analysis was performed to determine the thermal stability of the fabricated membranes. The degradation of the membranes was determined by weight loss over the temperature range from 0 °C to 800 °C, as shown in Figure 3. From the graph, it can be seen that the first thermal drop is at around 100 °C for all of the membranes, as this is due to the evaporation of the moisture at the surface of the membrane. The second thermal drop is between 100 °C to 430 °C, which resulted from the degradation of the functional group on the surface of the membranes, excluding the pristine membrane. Beyond 430 °C, steep drops are observed for all of the membranes. This shows the degradation of the main supportive polymer chain, PES. This result is in good agreement with Cheng and Chen [39]. M2 and M3 show improved thermal stability properties and obtained similar degradation rates with the inclusion of TiO_2_ and MWCNT. By incorporating both of the components, the thermal tolerance of M4 was further enhanced, which shifted to higher temperatures. A similar trend was also observed for M5, M6, and M7. However, functionalized membranes produce better thermal properties compared to non-functionalized membranes with lower percentages of the residual membrane at higher temperatures. This is because functionalized membranes form strong bonds with the components and are less vulnerable to heat degradation.

Membrane hydrophilicity plays an important role in fouling resistance, and previous research has suggested that organic components such as HA and protein usually exhibit lower fouling tendencies when using a more hydrophilic surface [40,41]. The contact angle of the prepared membranes is shown in Table 3. It can be clearly observed that the incorporation of the NPs led to the enhancement of the membrane hydrophilicity. For the M5, M6, and M7 membranes, the decline of the contact angle was approximately 14.80%, 10.50%, and 15.90%, respectively. Their improvement in terms of hydrophilicity might be due to the epoxy groups and the carboxyl groups of the functionalized NPs. This might also be a result of the lower interface energy of NPs and the movement of NPs towards the upper layer of the membrane matrix through a coagulation bath [42]. The highest water contact angle of 70.90° was obtained for the pure PES membrane (M1), signifying the lowest hydrophilicity, while the lowest contact angle of 55.00° was obtained by the M7 membrane indicating, the highest hydrophilicity. The reduction of the CA of the M7 membrane was due to the addition of fMWCNTs-TiO_2_, thus suggesting the attachment of the polar functional groups of fMWCNTs-TiO_2_, which increase the water diffusion within the polymer matrix. A similar trend was also observed in a study conducted on different concentrations of fMWCNTs by Rahimpour et al., which showed the reduction of the contact angle of the PES membrane compared to the fMWCNT/PES membrane [13]. 

**Figure 4:**
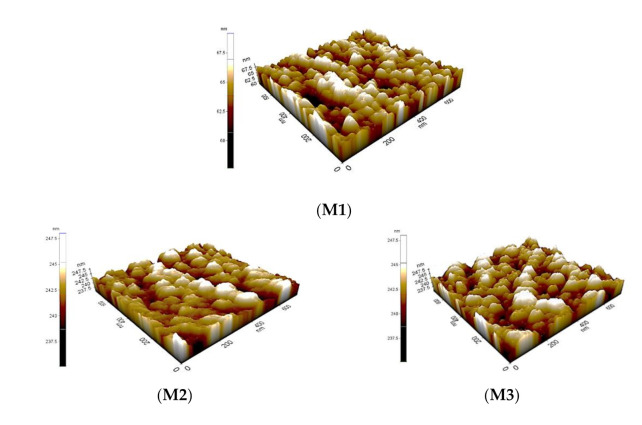

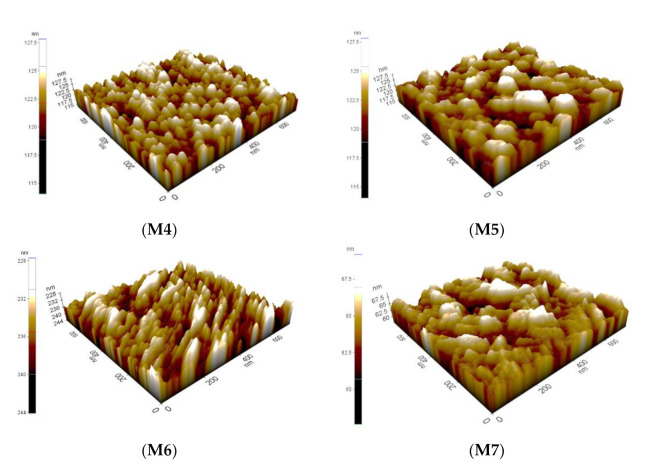
Atomic force microscopy (AFM) images of M1, M2, M3, M4, M5, M6 and M7 membrane.

Another important membrane characteristic influencing the desorption and/or adsorption of the solutes on the membrane surface and that affects membrane fouling propensity is the surface roughness. Atomic force microscopy (AFM) images of M1, M2, M3, M4, M5, M6 and M7 membrane are presented in Figure 4. The topography showed similar grain-like structures for all of membranes; however, there were differences in the heights across the membranes. The darkest regions signify the pores or valleys, while the brightest sites signify the maximum point on the surface of the membrane. The surface roughness values of the fabricated membranes are also shown in Table 3. The surface roughness of the unfunctionalized nanocomposite membranes (M2–M4) increases upon the addition of the particles but decreases upon the addition of the functionalized particles (M5–M7). This behavior could be due to the higher surface energy of the unfunctionalized particles and the weak interfacial interaction between the unfunctionalized particles with the PES polymer. This can also be observed from the SEM micrographs, which show that the functionalized nanocomposite membranes have less agglomerations than the unfunctionalized membranes. The surface roughness of the membrane containing fMWCNTs-TiO_2_ (Ra = 63.128 nm) was lower compared to the pristine membrane (Ra = 74.543 nm) and the other composite membranes. The decrease in surface roughness indicates that the peaks and depressions became smaller, consequently decreasing the mean pore radius. Thus, the M7 membrane is anticipated to have enhanced antifouling properties. This result is in good agreement with Razmjou et al., whose TiO_2_-modified blended membrane produced lower surface roughness compared to the pristine PES membrane [43].

Table 3 presents the mean pore radius, porosity, and overall membrane thickness of the prepared membranes. As shown, the pristine membrane (M1) has the thinnest thickness compared to the mixed-matrix membrane. The overall membrane thickness increased when the nanoparticles were incorporated. The incorporation of inorganic components will increase the viscosity of a doped solution, slowing down the H_2_O diffusion from the coagulation bath to the doped solution, and as a consequence, this increases the thickness of the skin-layer. Moreover, both kinetic and thermodynamic factors worked together to increase the thickness of the skin layer as the inorganic components were added. 

The porosity of membranes can be affected by many factors such as evaporation time and the coagulation process as well as solvent–polymer interaction. The porosity of the membranes is presented in Table 3. As shown, the overall porosity of the membranes follows the sequence of M6 > M5 > M3 > M7 > M4 > M2 > M1. In comparison with the pristine membrane, the porosity of all of the MMMs was higher. The high porosity of the membranes might be a result of formation of macrovoids and channels during phase the inversion process under the rapid movement of the water molecules. It may also be influenced by the improved hydrophilicity and polymer viscosity. Martin et al. [44] and Otitoju et al. [45] noted that greater hydrophilicity will reinforce the diffusional interaction between solvent and non-solvent, causing a lesser concentration of polymer during interphase between the doped solution and distilled water during the phase inversion process. The higher porosity of all of the MMMs may also play a vital role in the improvement of H_2_O permeability.

The mean pore radius of the fabricated MMMs is presented in Table 3. However, the mean pore radius slightly decreased upon the addition of fMWCNTs-TiO_2_. For the M3 membrane, a larger pore size was observed when the MWCNTs were added into the polymer solution. This could be the result of interfacial stress between the MWCNTs and the polymer, which builds up and then relaxes to form interfacial pores as a result of the shrinkage in the organic phase during the de-mixing process. As observed in Table 3, the membranes with a smooth surface had a smaller pore radius compared to the membranes with a rough surface. This is similar to observations by Yang et al. [46]. Among the other membranes, the M7 membrane shows the lowest pore size.

### 3.2. Membrane Performance

#### 3.2.1. Pure Water Flux (PWF) and Humic Acid Flux (HAF)

The results of the dead-end filtration tests for all of the fabricated membranes are displayed in Table 4. The results show that all of the MMMs present greater PWF ($J_{WF}$) values compared to the pure PES membranes (81.05 L/m^2^ h). This could be a result of the relatively high hydrophobic character of the pristine PES membranes. The $J_{WF}$ of the MMMs agrees with its spectacular hydrophilicity and mean pore size, as confirmed in Table 3. The membrane incorporated with MWCNT (M3) shows the maximum $J_{WF}$ of 107.46 L/m^2^ h, which is 1.3 times higher than the pristine membrane. In comparison to the other membranes, the improvement in the PWF of the M3 membrane couldt be due to the relatively large pore radius (51.62 nm). The $J_{WF}$ decreases in the order of M3 > M6 > M2 > M5 > M7 > M4 > M1. The decrease in $J_{WF}$ for the MMMs might be due to the change in the pore radius and the hydrophilicity (Table 3). In the case of HA flux, the M7 membrane presents the maximum permeate flux ($J_{HA}$) value of 62.01 L/m^2^ h. Even though the M7 membrane had a pore radius (29.02 nm) that was relatively smaller than that of the M3 membrane, the $J_{HA}$ of the M7 membrane was still superior. This might be a result of the deposition of the HA molecules in the valleys of the M3 membrane and as a consequence of $J_{HA}$ decline. The $J_{HA}$ decreases in the order of M7 > M6 > M5 > M3 > M2 > M4 > M1. The improvement in the $J_{WF}$ and $J_{HA}$ for all of the MMMs corresponds to the new H_2_O pathways and the variation in surface properties of the membranes (surface roughness, mean pore radius, and hydrophilicity) upon the addition of the NPs, which led to the decrease in the hydro-dynamic resistance through the selective layer, consequently improving membrane permeability. This observation is in tandem with an observation by Boshrouyeh et al. [47] and Safarpour et al. [48]. The $J_{HA}$ of membrane incorporated with fMWCNTs-TiO_2_ (M7) was higher than that of the other MMMs (M2–M6), and due to its more hydrophilic nature coupled with its high porosity, the pristine membrane (M1) thus provided less resistance to water permeability. Since the hydrophilicity of the composite membrane (M7) was higher than other membranes, the improved hydrophilicity will cause interfacial resistance to decline, promoting the passage of H_2_O molecules through the pores of the membrane (via reducing threshold pressure for the transportation of H_2_O across the membrane) and will thus increase the water permeability [49]. The result in this work has shown that the incorporation of fMWCNTs-TiO_2_ significantly helps to enhance H_2_O permeability and thus demonstrates the credibility of the M7 membrane.

#### 3.2.2. Humic Acid (HA) Rejection 

Table 4 also presents humic acid rejection for all of the membranes. The order of humic acid rejection is as follows: M7 > M5 > M6 > M2 > M1 > M4 > M3. As observed, the highest HA rejection was obtained for the M2, M5, M6, and M7 membranes. The composite membrane (M3) presents the lowest HA rejection of 72.71%, whereas the M7 membrane presents the highest HA rejection of 92.00%. Although the M3 membrane has the highest PWF, this membrane presents the lowest HA rejection among all of the MMMs. The higher pore size of the M3 membrane will influence the easy passage of water as well as HA molecules through the membrane surface. Furthermore, the percentage of HA rejected from the membrane with functionalized particles was higher than that of the unfunctionalized membranes, implying that the selectivity of the MMM was also improved via the addition of functionalized NPs, with the membranes functionalized with fMWCNTs-TiO_2_ displaying the highest HAR. Among all of the composite membranes, M7 presents a better performance improvement without compromising HAR, owing to the smallest mean pore size and improved hydrophilicity, which could influence its antifouling characteristics. Pore size reduction will have a significant influence on the steric hindrance effect in the membrane.

#### 3.2.3. Membrane Fouling Analysis

**Figure 5:**
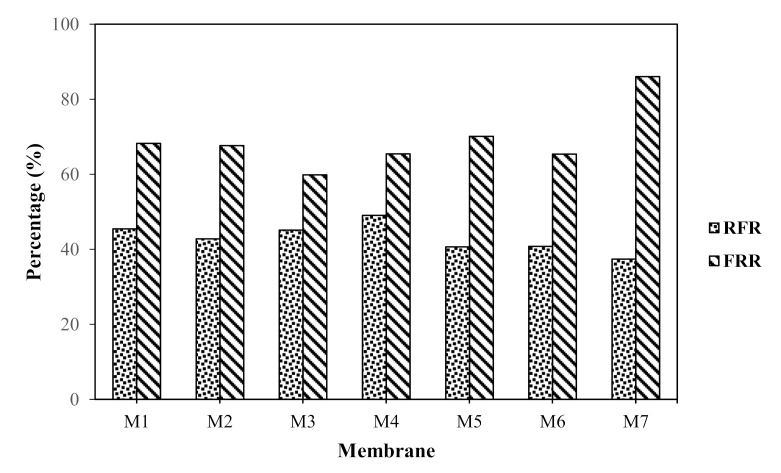
Flux recovery ratio (FRR) and relative flux reduction (RFR) of M1, M2, M3, M4, M5 M6 and M7 membrane.

In water purification, fouling is one major drawback affecting membrane performance due to the hydrophobic nature of prepared membranes. To observe the antifouling capabilities, the FRR and RFR of the membranes are presented in Figure 5. In general, the higher the FRR, the higher the recycling property and resistance to HA fouling. The FRR is in the order of M7 > M5 > M1 > M2 > M4 > M6 > M3. Among all of the fabricated membranes, the M7 membrane presents the highest FRR, indicating high cleaning efficiency. The M3 membrane presents the lowest FRR value, which signifies its high risk to membrane fouling. In the case of the M7 membrane, its better FRR could be due to improved surface hydrophilicity and the lowest membrane roughness. First, a lower roughness means less HA adsorption. Second, It has been well established that a more hydrophilic membrane will have a lower fouling tendency [50,51]. A membrane with a better hydrophilic character will weaken the hydrophobic interaction between the membrane surface and the foulant, enabling the foulant that is adsorbed on membrane surface to be easily washed away using hydraulic method of cleaning. 

Moreover, the introduction of fMWCNTs-TiO_2_ on the surface of the membrane will cause a steric hindrance effect, which might prevent HA from making contact with the membrane [52,53]. This result indicates that the MMM incorporated with fMWCNTs-TiO_2_ NPs was easier to clean than the MMM and that it imparts resistance to HA adsorption. This result also confirms the optimistic efficaciousness of fMWCNTs-TiO_2_ NPs, which will thus reduce the cost of maintenance as well as the cost of sustainable filtration materials. 

The M4 membrane presents the highest RFR value of 49.08%, indicating that the membrane suffered from a serious flux decline due to fouling caused by HA. The high RFR of the M4 membrane might be the result of its highest surface roughness. The RFR reduced to 45.50%, 45.15%, 42.80%, 40.80%, 40.70%, and 37.39% for M1, M3, M2, M6, M5, and M7, respectively (Figure 5). The RFR of the M7 membrane was the lowest compared to that of the pristine and other composite membranes, signifying that the M7 membrane had better cleaning efficiency. Surface roughness and hydrophilicity are important factors undermining the fouling behavior of membranes. Membrane fouling tends to be higher when the surface becomes rougher or more hydrophobic. This situation is vice versa when the membrane surface is more hydrophilic. As previously discussed, the membrane hydrophilicity was significantly improved, and the surface roughness was reduced after with the incorporation of fMWCNTs-TiO_2_. The improved anti-fouling capability of the M7 membrane reveals the impact of reduced surface roughness and enhanced hydrophilicity.

The results in this work indicate that the f-MWCNTs-TiO_2_ membranes provide sufficient HA rejection efficiency and considerable permeation flux. In Table 1, a comparative study of this work to other f-MWCNTs membranes in previous works is summarized. As observed, the f-MWCNTs-TiO_2_ membrane presents a reasonable and superior performance in comparison with other works in the literature. Based on the performance in the current work, it can be concluded that the f-MWCNTs-TiO_2_ membrane possesses great potential for the treatment of HA wastewater.

## 4. Conclusions

In this work, MWCNT particles were successfully functionalized using co-precipitation and were then loaded with TiO_2_ particles to prepare a hybrid membrane for the removal of humic acid (HA). The microstructure of the prepared membrane was characterized by SEM, AFM, FTIR, water contact angle, pore size, porosity, and thickness as well as their performance for HA removal were characterized by pure water flux (PWF), HA flux (HAF), HA rejection, and fouling resistance. It was observed that the sensibility of the prepared membrane to fouling is alleviated with a decrease in the roughness of the surface. The results from this study (PWF: 99.05 L/m^2^ h, HAF: 62.01 L/m^2^ h, HA rejection: 92%, RFR: 37.40%, FRR: 86.02%) determined that fMWCNTs loaded TiO_2_ nanoparticles is a prodigious anti-fouling material, which may lead to new applications for membranes for use in the high-quality removal of humic acid.

## Figures and Tables

**Table 1:** Polyethersulfone-based functionalized MWCNTs membranes for wastewater treatment.

Membranes	Performance				Ref.
	Pure Water Flux	Permeate Flux	Rejection	Fouling	
MWCNTs-COOH	-	BSA (31.48 L/m^2^ h)	-	Nominalized irreversible fouling: 0.08	[20]
0.025 wt% MWCNTs-COOH	-	-	BSA (>99%)	FRR for synthetic municipal wastewater (81.4 ± 3.5%), FRR for BSA (89.3 ± 2.1%)	[21]
0.5 wt% NH_2_ functionalization of MWCNTs	~150 L/m^2^ h	~32 L/m^2^ h	0.5 g/L BSA (>87%)	-	[13]
PCA-0.1 wt% MWCNT	-	20–25 L/m^2^ h	-	FRR for whey protein (95%)	[22]
0.5 wt% ZnO/COOH-MWCNTs	-	16.7 L/m^2^ h	Powder milk (88.6%), Dye removal (>90%)	Irreversible resistance (11.4%)	[23]
0.5 wt% HPEI/COOH- MWCNTs/Fe-Cu	-	26.3 ± 1.3 L/m^2^ h	2,4,6-TCP removal (99.4%)	FRR for BSA (>90%),	[24]
0.05% MWCNT-PNIPAAm	-	~50 kg/m^2^ h	COD (90%)	Total fouling ratio49.98%, FRR (~99.9%)	[25]
0.5 wt% β-CD/MWCNTs	-	~21.5 L/m^2^ h	Dye rejection (>92%)	Irreversible resistance (11.1%), FRR (84%),	[26]
0.1 g Ni@MWCNTs with magnetic field	1060.93 L/m^2^ h	-	-	FRR for BSA (67.89%), SA (85.53%), YE (60.28%), HA (90.12%).	[27]
NH_2_-MWCNTs	-	90.85 L/m^2^ h	Oil rejection (>99%)	Total fouling ratio 22.35%, FRR of ~95.73%.	[28]
0.05 wt% Oxidized-MWCNTs	553 L/m^2^ h	-	BSA (>99.9%)	FRR of >90%.	[29]
1.0 wt% Fe-Ag/Acid treated-MWCNTs	-	36.9 L/m^2^ h	Cr^6+^ ion rejection (93.74%)	Fouling resistance (94.98%)	[30]
0.4 wt% TETA-functionalized MWCNTs	84.35 L/m^2^ h	-	BSA (93.1%), Rhodamine B rejection (99.23%) Orange G rejection (82.13%) Crystal violet rejection (98.43%) Indigo rejection (87.12%)	Irreversible fouling (6.88%)	[31]
0.1 wt% NH_2_-MWCNTs	-	84 L/m^2^ h	BSA (~60%)	FRR for activated sludge suspension (89.7%)	[32]
Acid treated-MWCNTs	~72.2 L/m^2^ h	-	BSA (90%)	-	[33]
f-MWCNTs	99.05 L/m^2^ h	62.01 L/m^2^ h	HA (92%)	FRR (86.02%)	This work

*Abbreviations: Amino (NH_2_); amino-functionalized multi-wall carbon nanotubes (N-MWCNTs); bovine serum albumin (BSA); carboxylic groups (-COOH); chromium (Cr); copper (Cu); humic acid (HA); hyperbranched polycitricacid (PCA); hyperbranched polyethyleneimine (HPEI); iron (Fe); multi-wall carbon nanotubes (MWCNTs); nickel (Ni); N-isopropyle acryleamide (NIPAAm); silver (Ag); sodium alginate (SA); triethylenetetramine (TETA); yeast (YE); zinc oxide (ZnO); β-cyclodextrin (β-CD).

**Table 2:** The composition of the doped solution.

Label	Membrane Components	PES (wt.%)	DMAc (wt.%)	Nanoparticles				
				TiO_2_ (wt.%)	MWCNTs (wt.%)	fTiO_2_ (wt.%)	fMWCNTs (wt.%)	fMWCNTs-TiO_2_ (wt.%)
M1	PES membrane	17.0	83.0	-	-	-	-	-
M2	PES + TiO_2_ membrane	17.0	82.0	1	-	-	-	-
M3	PES + MWCNTs membrane	17.0	82.0	-	1	-	-	-
M4	PES + TiO_2_/MWCNTs membrane	17.0	82.0	0.5	0.5	-	-	-
M5	PES + fTiO_2_ membrane	17.0	82.0	-	-	1	-	-
M6	PES + fMWCNTs membrane	17.0	82.0	-	-	-	1	-
M7	PES + fMWCNTs-TiO_2_ membrane	17.0	82.0	-	-	-	-	1

**Table 3:** The overall membrane thickness, roughness, porosity, and mean pore radius of membranes.

Membranes	Thickness (µm)	Porosity (%)	Mean Pore Radius (nm)	Surface Roughness (nm)	CA (°)
M1	180 ± 1.0	57.38 ± 0.2	34.65 ± 0.2	74.543	70.90
M2	200 ± 1.0	64.90 ± 0.2	32.39 ± 0.4	77.591	59.23
M3	230 ± 1.0	69.37 ± 0.2	51.63 ± 0.4	79.972	65.12
M4	210 ± 1.0	67.55 ± 0.2	31.45 ± 0.3	82.673	59.82
M5	200 ± 1.0	70.29 ± 0.2	33.57 ± 0.4	66.732	56.10
M6	210 ± 1.0	70.84 ± 0.2	34.92 ± 0.2	65.261	60.40
M7	200 ± 1.0	69.09 ± 0.2	29.02 ± 0.2	63.128	55.00

**Table 4:** J_WF_, J_HA,_ J_WF2_, and humic acid (HA) rejection of the fabricated membranes.

Membranes	$J_{WF}\;(L/m^{2}\;h)$	$J_{HA}\;(L/m^{2}\;h)$	$J_{WF2}\;(L/m^{2}\;h)$	Rejection (%)
M1	81.05	44.17	55.34	76.79
M2	104.41	58.01	68.60	77.12
M3	107.46	58.94	64.35	72.71
M4	91.15	46.41	59.65	74.48
M5	103.27	61.24	72.40	82.93
M6	104.70	61.98	68.49	82.71
M7	99.05	62.01	85.21	92.00

## Data Availability

The data presented in this study are openly available in the results and discussion section of this study.
